# Relationships between alpha oscillations during speech preparation and the listener N400 ERP to the produced speech

**DOI:** 10.1038/s41598-018-31038-9

**Published:** 2018-08-27

**Authors:** David A. Bridwell, Sarah Henderson, Marieke Sorge, Sergey Plis, Vince D. Calhoun

**Affiliations:** 1 The Mind Research Network, Albuquerque, NM USA; 2 Department of Electrical and Computer Engineering, University of New Mexico, Albuquerque, NM USA

## Abstract

While previous studies separately demonstrate EEG spectral modulations during speech preparation and ERP responses to the listened speech, it is unclear whether these responses are related on a trial-by-trial basis between a speaker and listener. In order to determine whether these responses are related in real-time, Electroencephalography (EEG) responses were measured simultaneously within a speaker and listener using a 24 electrode Mobile EEG system (18 participants; 9 pairs) during a sentence completion task. Each trial consisted of a sentence prompt with an incomplete ending (e.g. “I took my dog for a ____”). The speaker was instructed to fill in the ending with something expected (e.g. “walk”) (40 trials) or unexpected (e.g. “drink”) (40 trials). The other participant listened to the speaker throughout the block. We found that lower alpha band activity was reduced when individuals prepared unexpected sentence endings compared to expected sentence endings. Greater reductions in the speaker’s lower alpha activity during response preparation were correlated with a more negative N400 response in the listener to the unexpected word. These findings demonstrate that alpha suppression and the N400 ERP effect are present within a hyperscanning context and they are correlated between the speaker and listener during sentence completion.

## Introduction

During communication, the speaker is tasked with generating a sequence of words which are semantically logical and which follow syntactic structure. The listener is tasked with incorporating the pragmatics, prosody, semantics and syntax of the listened speech into meaning^1^. In order to accomplish these tasks, the listener appears to track these speech features, generate predictions for upcoming speech, and engage in further processing when predictions are violated^2–5^.

These aspects of speech processing are likely supported by the series of cortical oscillatory and event-related potential (ERP) responses that appear during speech, illuminating the neural mechanisms that likely support speech and language comprehension. The most prominent language-related ERP peaks occur approximately 200 ms, 400 ms, and 600 ms following the onset of speech violations. The early negative peak at ~200 ms appears to correspond to instances where the earliest phonological unit does not align with the phonological unit that is expected^6,7^. The negative peak at 400 ms, termed the N400, appears sensitive to violations of the expected semantic meaning of a word (given the preceding context)^1,8^, and the positive peak at 600 ms (termed the P600) is primarily modulated by violations in syntax (i.e. grammatical structure)^9^.

Importantly, the N400 shows a graded variation in response amplitude as a function of the surprise of unexpected words^2,8,10^, and areas involved in the N400 appear sensitive to the degree in which verbs constrain subsequent nouns (e.g. a greater response to the verb “drive”, which predicts “car”, than to the verb “get” which is less predictive of the noun that will follow)^5^. The N400 thus appears to index semantic prediction, consistent with predictive coding theories which posit that higher order contextual areas transfer predictions to early sensory areas in anticipation of incoming sensory stimuli^4,11,12^. The P600, in contrast, does not appear to be modulated by predictive processes directly, but instead appears to reflect repair processes that follow syntactic violations^13,14^.

An important factor that may mediate the presence and magnitude of these ERP components is the complexity of the speech stimulus and the larger social context. For example, the presence of the N200 and the latency of the N400 each depend on whether words are presented with pauses or continuously, as in natural speech^15^. In addition, the N400 effect is present when anomalies appear at a broader level of discourse than a single sentence^16^, and the effect is modulated by the social context of having another listener present^17^. However, since these ERP effects have been primarily studied with words presented visually on a computer screen or aurally through speakers, their presence has yet to be examined in the natural communicative context of listening to a speaker constructing semantic violations in real time. Measuring EEG responses within the speaker and listener simultaneously will help identify the subset of language ERP effects and speaker EEG effects that may be related within a more natural speaking-listening context.

Differences in EEG spectra during speech preparation likely reflect the sensitivity of EEG oscillations to cognitively demanding tasks. These differences are generally specific to the alpha frequency band (8–12 Hz), which shows robust increases when individuals close their eyes, and decreases when individuals engage in visual tasks^18^. These changes in alpha activity are thought to represent changes in the “idle” state of the visual system, with increases in alpha activity potentially reflecting a “disengagement” of the visual system from visual inputs and the rest of the brain^19,20^. Correspondingly, reductions in alpha activity are related to improvements in detecting visual targets^21–23^, motivating its contribution speech preparation within visual sentence completion tasks.

The suppression of alpha activity that occurs during demanding tasks like speech preparation, and N200, N400 and P600 ERP components to speech violations, have primarily been studied within experimentally constrained contexts, which limit the social or ecological relevance of the task. Within the present study, we leverage these two phenomena within an EEG hyperscanning context to determine whether modulations in the speaker’s alpha activity when they prepare an unexpected word are related to trial-by-trial modulations in the listeners N400 ERP response to the semantic incongruity.

The speaker is asked to complete a sentence with a “humorous” or “serious” word (e.g. “I took my dog for a drink”, or “I took my dog for a walk”, respectively). The “humorous” instruction encourages individuals to come up with an unexpected sentence ending, which, due to its greater difficulty than the “serious” instruction, will likely be associated with reductions in the speakers EEG alpha band activity. Reductions in the alpha band response will correspond to a greater ability to focus on the task, leading to the successful generation of unexpected sentence endings. We predict that these unexpected sentence endings will correspond to more negative amplitudes of the N400 component, since it is modulated by semantic incongruity. Thus, we predict that reductions in single trial alpha amplitudes within the speaker during response preparation will correspond to more negative N400 amplitudes in the listener to the spoken word.

## Materials and Methods

### Participants

Ten pairs of individuals were recruited to participate in a single session (20 participants). Four pairs were female-female, 5 pairs were male-male, and the 1 pair was female-male (mean age: 31.60; standard deviation (std): 6.69). One participants data was discarded due to experimental error, and another participants data was discarded due to an excessive number of bad channels (18 of 24), reducing the number of participants included in the analysis to 18. Each individual had normal audition and had no family history of mental illness. All participants were enrolled in protocols approved by the University of New Mexico Institutional Review Board (HRRC/MCIRB). All procedures were explained to the participants before the study, and written informed consent was obtained prior to the session at the Mind Research Network. The procedures were performed in accordance with ethical guidelines and regulations.

### Experimental Design and Stimuli

We used the SMARTING Streamer (the software interface for mBrainTrain’s EEG amplifier) to collect data on a mobile phone (speaker) or laptop (listener). The amplifier is connected to the device with Bluetooth manager BlueSoleil (laptop) or through built-in Bluetooth (mobile phone). Experimental stimuli were presented on a SONY Xperia Z Ultra mobile phone using OpenSesame experimental software (http://osdoc.cogsci.nl/)^24^.

The phone was placed ~56 cm in front of the speaker. Experimental triggers were sent to SMARTING at the onset of the instruction, the onset of the sentence, and when the sentence changed from white to green. The speaker’s audio was acquired through a headset microphone connected to the laptop and audio signals were acquired in synch with the listeners EEG using Lab Streaming Layer (LSL) (https://github.com/sccn/labstreaminglayer).

**Figure 1:**
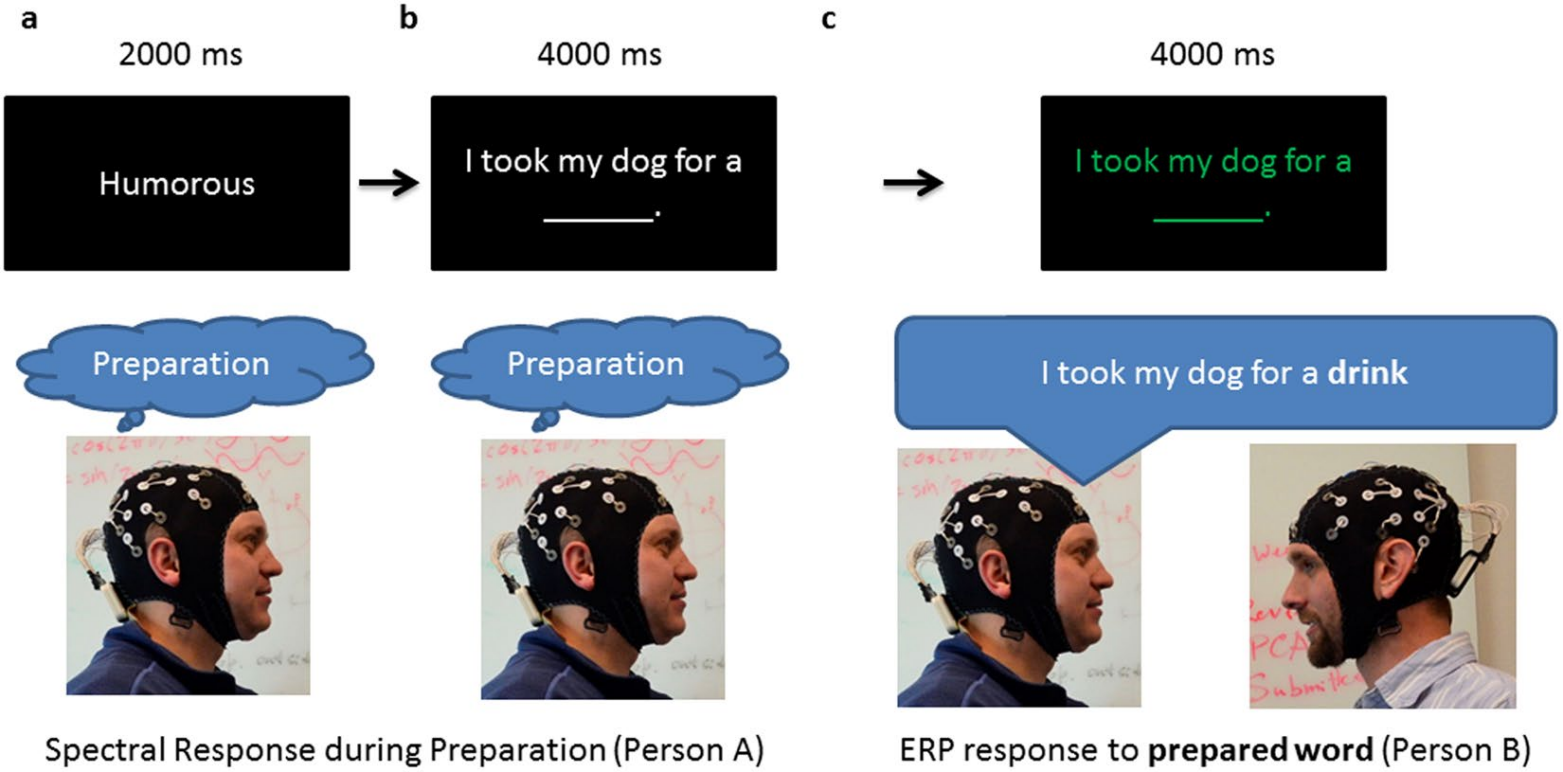
Task Design. The speaker (person A) is instructed to fill in a sentence with a humorous (i.e. unexpected) ending (**a**) (2000 ms), followed by a sentence with an open ending (**b**) (e.g. “I took my dog for a ____” (4000 ms). The speaker is prompted to read the sentence aloud to the listener when it turns green (**c**), including the unexpected ending (e.g. “I took my dog for a party”). As denoted underneath the pictures of the participants, the spectral responses of the listener during initial preparation (**a**) are compared with the ERP response to the unexpected word within the listener (**c**). The “humorous” prompt in (**a**) is replaced by the word “serious” during the “expected” condition, when the speaker prepares the expected ending of the sentence (i.e. “I took my dog for a walk”).

The session was comprised of two 13 m 36 s blocks, with 80 trials per block. For the first block, one subject was assigned the role of “speaker” and the other subject was assigned the role of “listener”. These roles were reversed in the second block, and the same set of 40 sentences were used in each of the two conditions (see supplementary material). Each trial began with a 200 ms fixation, followed by the prompt “serious” or “humorous” to instruct the participant to produce expected or unexpected sentence endings, respectively (see supplementary material). The prompt appeared for 2000 ms and was displayed in white on a black background. The prompt was followed by a sentence with an incomplete ending (e.g. “I took my dog for a ____”) (4000 ms). After 4 s., the sentence turned green, and the speaker read the sentence aloud, including the filled in word. The sentence remained green for 4 seconds until the next trial began (Fig. 1). The listener sat across from the speaker and was instructed to listen to the speaker throughout the block.

### EEG Acquisition and Preprocessing

EEG data was collected using a 24-channel SMARTING amp (mBrainTrain http://www.mbraintrain.com) (sample rate = 500 Hz) and an EasyCap EEG cap. EEG activity was recorded using sintered Ag–AgCl electrodes placed according to the 10–20 International System, with CPz and Pz added (the common mode sense (CMS) and driven right leg (DRL) electrodes were located on FCz and Fpz, respectively). Electrode impedances were kept below 10 kΩ.

EEG preprocessing was conducted in Matlab (http://www.mathworks.com) using custom functions, built-in functions, and the EEGLAB toolbox (http://sccn.ucsd.edu/eeglab). Blink artifacts were attenuated by conducting a temporal ICA decomposition on the individual recordings (extended Infomax algorithm in EEGLAB)^25,26^. Artifactual components were identified by visual inspection of the component time-course, topographic distribution, and frequency spectrum and removed from the back reconstructed time-course^27^. On average, 1.26 components were removed from the speakers EEG (min: 1; max: 2), and 1.37 components were removed from the listeners EEG (min: 1; max: 5).

The reconstructed EEG data was linearly detrended and re-referenced to the average of the mastoids. Bad channels were identified based on the data distribution and variance of channels, as implemented in EEGLAB’s pop_rejchan function^28^ and the FASTER toolbox^29^, and spherically interpolated. On average, 1.53 channels were removed (std = 2.61) from the speakers EEG, and 2.42 channels were removed from the listeners EEG (std = 4.40).

The word onsets were manually identified by adjusting the length of the sentence in 10 ms increments until the first phoneme of the final word was aurally perceptible. Using this approach, the onset of the final word is clearly present around 0 ms within the average speech envelope of each condition (see Supplementary Fig. 1). The listeners EEG signals were segmented within the interval −200 ms and 1000 ms surrounding the onset of the expected or unexpected word. Epochs were removed if any Bluetooth packets were lost within the interval or if the speaker was unable to fill in the sentence with an unexpected word. Artifactual epochs were identified using the automatic artifact epoch detection algorithm in EEGLAB (function: *pop_autoreg*) and excluded from subsequent analysis. On average, 9.32 out of 80 epochs met criteria for removal (std = 6.20) across listeners. The same criteria was used for identifying bad epochs within the −200–1000 ms interval following the “humorous” or “serious” prompt within the speakers data, and 13.84 out of 80 epochs met criteria for removal (std = 6.91) per subject. Epochs removed from the speaker were also removed from the listeners data (and vice versa), bringing the average number of epochs excluded to 14.05 (std = 6.84).

### Listener ERP Amplitudes

Responses were averaged across central-parietal electrodes since previous studies demonstrate a robust negative response over these electrodes 400 ms after semantically inappropriate words^1,8^. Epochs were averaged across participants and conditions and the time point of the positive and negative ERP peaks were identified. (Averaging across conditions ensures that the choice of time point is unbiased with respect to differences between conditions.) The average amplitude at the peak time point was computed for individual subjects for expected and unexpected trials to determine whether the absolute value of ERP amplitudes are greater for the unexpected sentence endings, consistent with previous studies.

### Speaker wavelet power

The speaker’s EEG was decomposed into frequencies with wavelet analysis in order to examine differences in spectral responses during response preparation (i.e. following the “serious” or “humorous” instruction). Wavelet transformation was conducted with the continuous wavelet transform function in Matlab version 8.3 (*cwt*) using the complex Morlet wavelet (bandwidth parameter: *f*_*b*_ = 0.5)), with frequencies centered on 2, 6, 8, 12, 20 and 40 Hz. These frequencies represent the predominant frequency bands of EEG (i.e. delta, theta, lower alpha, upper alpha, beta, and gamma). Wavelet power was computed by taking the variance of the complex valued wavelet coefficients across trials, then computing their absolute value. This returns the magnitude of the speaker’s non-phase locked response to the “humorous” or “serious” prompt, within each frequency band.

We anticipated that lower alpha band responses would be reduced for the “unexpected” condition compared to the “expected” condition due to the greater attentional engagement required to generate an unexpected sentence ending. These results would be consistent with the robust decrease in 8 Hz alpha band responses observed during demanding tasks, and the increase in 8 Hz band responses observed during relaxation^18^. Responses were averaged across parietal electrodes (P7, P3, Pz, PO2, P4, and P8) since previous studies demonstrate robust alpha band responses over these regions. Epochs were averaged across participants and trials and the time point of the positive peak alpha band peak was identified. (Averaging across conditions ensures that the choice of time point was unbiased with respect to differences between conditions.) The average power at the peak time point was computed for individual subjects for expected and unexpected trials to determine whether alpha power was reduced following the “humorous” prompt (compared to the “serious” prompt).

### Relationships between speaker spectra and the listener ERP

Single trial listener ERP amplitudes were averaged across central-parietal electrodes separately for the negative and positive peak time points (identified from the aggregate data, as described in *Listener ERP amplitudes*). Single trial speaker power values were computed by averaging the complex valued wavelet coefficients across parietal electrodes (P7, P3, Pz, PO2, P4, and P8) at the peak time point (identified from the aggregate data, as described in *Speaker Wavelet Power*), and taking the absolute value. The speaker’s single-trial alpha band amplitudes were correlated with the corresponding listener single-trial N400 amplitudes for all unexpected trials, in order to determine whether reductions in the speakers alpha band response during unexpected word preparation are related to the listeners N400 response to the spoken word.

### Statistical analysis

One statistical test (T-test) was conducted to examine differences in wavelet energy between the “unexpected” and “expected” conditions within the speaker, and two statistical tests (T-tests) were conducted to examine differences in the listeners positive and negative ERP peaks between these two conditions. These tests were conducted to replicate well-established findings (i.e. an N400 response to semantically inappropriate words^8^, and a reduction in alpha during attentionally demanding tasks)^18^ and are reported as significant if they pass the uncorrected threshold of p < 0.05.

The relationship between peak amplitudes within the speaker and listener were examined for all frequency bands in order to determine whether relationships were restricted to the alpha band, as hypothesized. Twelve (6 frequencies ×2 peaks) one sample T-tests were conducted to determine whether Fisher z-transformed Pearson correlation coefficients between speaker and listener were significantly greater than zero. These statistical tests are reported as “significant” if they pass Holm–Bonferroni correction for the 12 comparisons (alpha = 0.05) (i.e., the 12 uncorrected p-values are ordered and the lowest p-value is significant if it is below 0.05/12 = 0.0042, the second lowest is significant if it is below 0.05/11 = 0.0045, and so on^30^).

## Results

### Behavior

During the “unexpected” condition, different sentence endings are likely to have different cloze probabilities, i.e. the percentage of subjects who use that word to end a sentence. Since the sentence endings are determined in advance in traditional N400 experiments, sentence endings can be obtained for a separate set of subjects prior to the experiment, and final words can be selected such that the cloze probability is known in advance. Since the sentence endings are not fixed advance in the present study, we calculate close probability as the percentage of times the *most frequently used* sentence ending was repeated. For example, individuals said “I took my dog for a walk” for 14 out of 15 trials in the “expected” condition, giving a cloze probability of 0.933 for this sentence. In the “unexpected” condition, the participants each gave unique answers to this sentence, including “I took my dog for a drink”, “I took my dog for a cat” and “I took my dog for a party”.

The cloze probabilities are reported for the most frequent word for each sentence and condition within the Supplementary Table. According to the guidelines of^31^, in the “expected” condition we found that 33 out of the 40 sentences have high close probability (i.e. from 0.67 to 1.00) and 7 out of the 40 sentences have medium close probability (i.e. between 0.34 and 0.66).

Among the 40 sentences the most common sentence ending was repeated 82.4% of the time on average (std = 0.17;min = 40.0%;max = 100%) for the “expected” condition, and 15.5% of the time (std = 0.07; min = 05.9%; max = 33.3%) for the “unexpected” condition. These results indicate that the sentence endings were well constrained^31^.

### Spectral differences during response preparation

**Figure 2:**
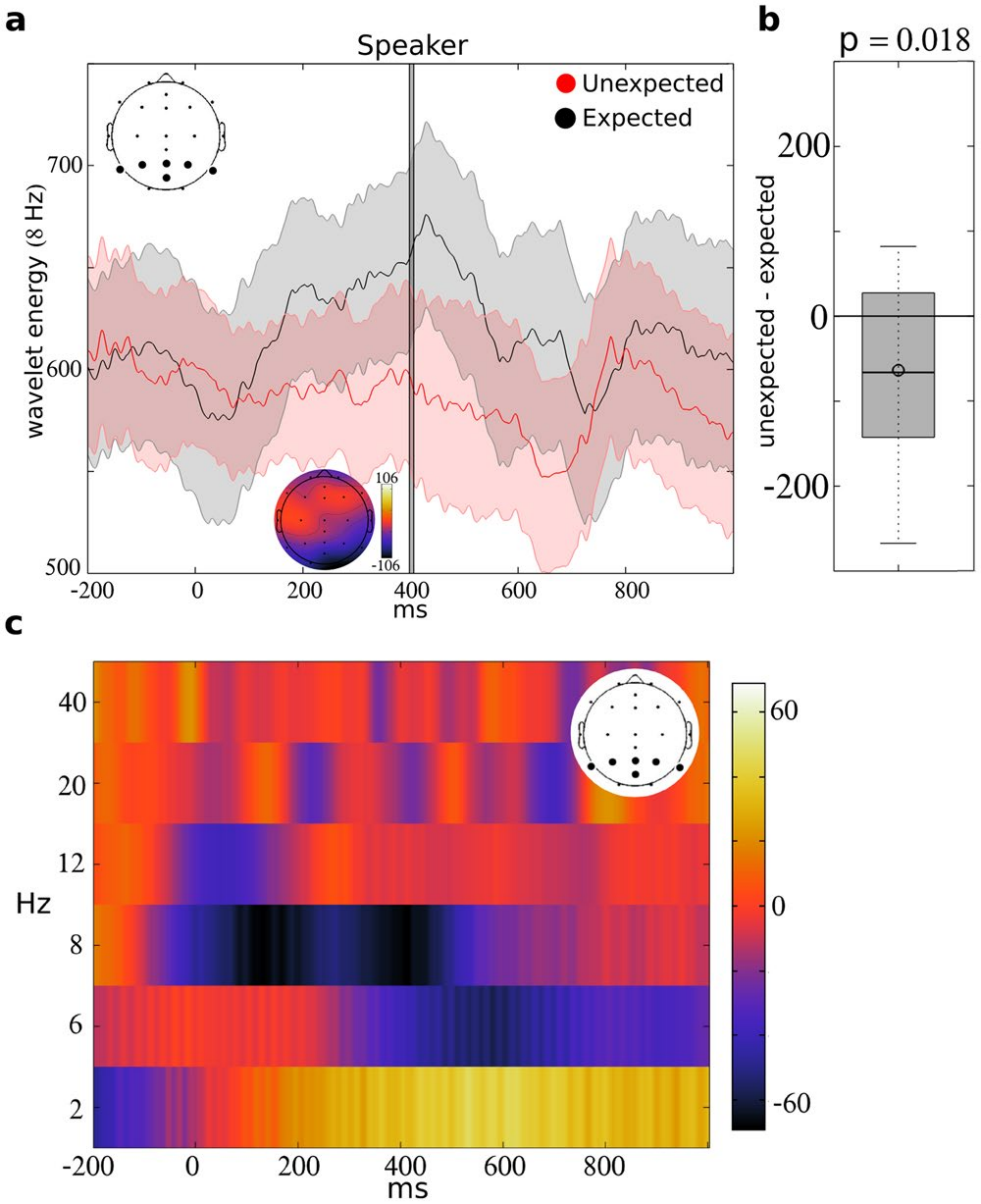
Difference in the speaker’s spectral energy during “expected” and “unexpected” conditions. The wavelet energy within the alpha band (centered on 8 Hz) is indicated for the speaker in (**a**) following the “expected” (black) or “unexpected” (red) prompt. Responses were averaged across parietal electrodes P7, P3, Pz, PO2, P4, and P8, as indicated by the plot on the upper left. The gray bar at 402 ms indicates the peak calculated from the overall average, and the topographic plot indicates the difference (unexpected minus expected) in average alpha energy at that time point across electrodes. The adjacent boxplot (**b**) indicates the distribution of individual differences (unexpected minus expected) in wavelet energy at that point. These values significantly differed from zero (p = 0.018). The difference in the speaker’s average spectral energy during response preparation (“unexpected” minus “expected”) is indicated for 2, 6, 8, 12, 20 and 40 Hz (y-axis) in (**c**). The onset of the “expected” or “unexpected” prompt is indicated by 0 ms (x-axis). Responses are averaged across parietal electrodes P7, P3, Pz, P02, P4, and P8, as indicated by the plot on the upper right.

Wavelet analysis was conducted to decompose the EEG into frequencies, and the difference in spectral energy was examined during response preparation. As demonstrated in Fig. 2a, lower alpha band energy increased in the interval from ~200 to 600 ms after the prompt in the “expected” condition, while alpha energy appears unaltered within this interval in the “unexpected” condition. The average peak wavelet energy was 657.83 in the “expected” condition and 593.93 in the “unexpected” condition, indicating a 09.71% reduction in response which was statistically significant (T = 2.61; p = 0.018; degrees of freedom (df) = 17) (Fig. 2b). Consistent with our hypothesis, the largest differences between the conditions was present within the lower alpha band (centered on 8 Hz) compared to the other frequencies (2, 6, 12, 20 and 40 Hz) (Fig. 2c). This finding supports the notion that the “unexpected” condition is more engaging (i.e. requires more resources) than the “expected” condition. Correspondingly, we hypothesize that greater reductions in the alpha band response will correspond to a greater ability to produce an unexpected word.

### ERP differences to the spoken word

**Figure 3:**
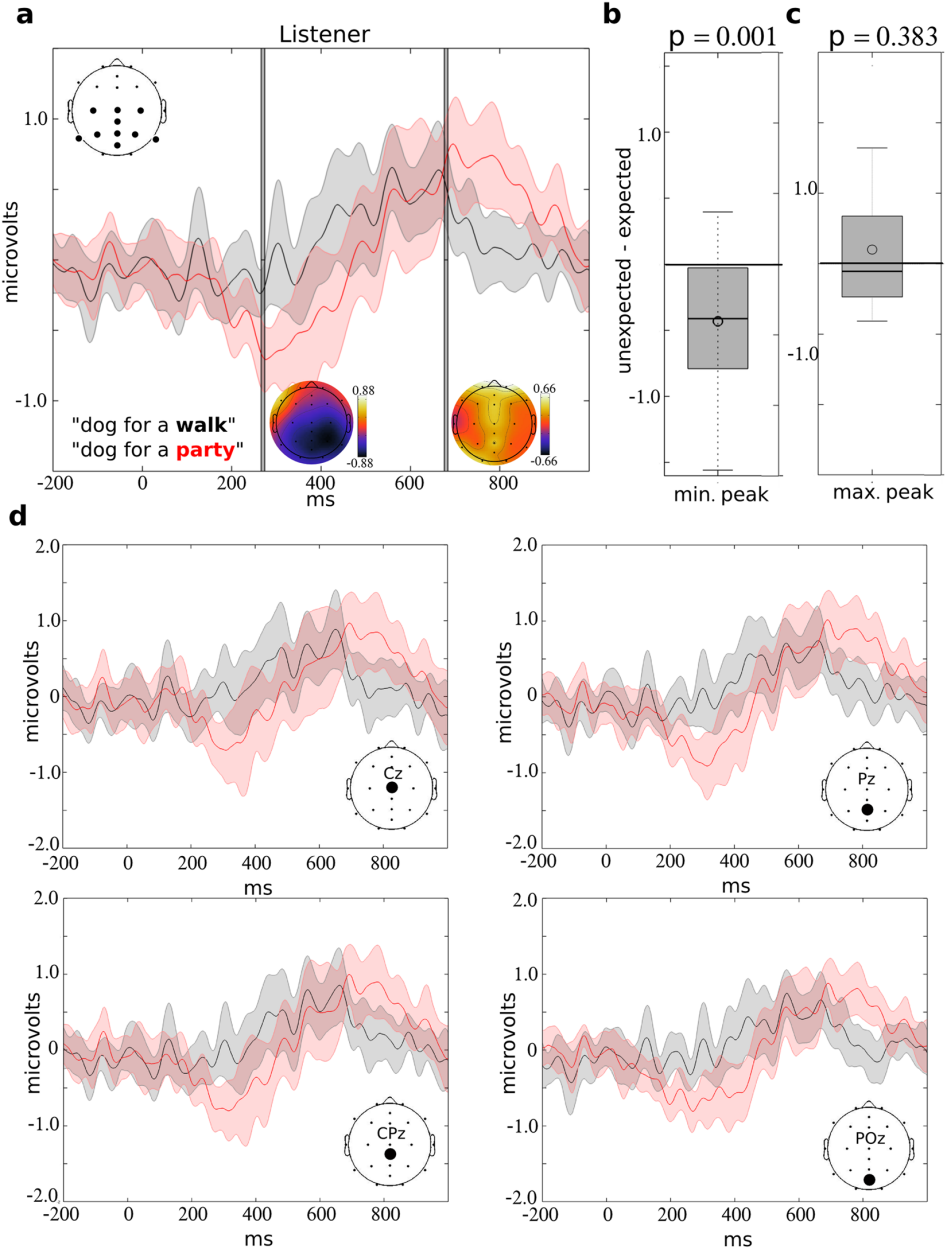
Average listener ERP to expected and unexpected words. The ERP response within the listener is indicated in (**a**) following the onset of the unexpected (red) or expected (black) word. Responses were averaged across central-parietal electrodes P7, P3, Pz, PO2, P4, P8, CPz, C3, Cz, and C4, as indicated by the plot on the upper left. The gray bars at 270 and 680 ms indicate the minimum and maximum peaks identified from the overall average, and the adjacent topographic maps indicate the difference (unexpected minus expected) in average amplitudes across electrodes at each time point. The boxplots indicate the distribution of individual differences (unexpected minus expected) in ERP responses for the minimum (**b**) and maximum (**c**) peaks. These values significantly differed from zero for the negative peak (p = 0.001) but did not reach statistical significance for the positive peak (p = 0.383). The width of shading around the red and black lines represents the 95% confidence interval of the mean. Within (**d**) the average ERP is indicated for Cz, CPz, Pz, and P0z electrodes (see topographic plot) for the expected (in black) and unexpected (in red) conditions. The width of shading around the red and black lines represents the 95% confidence interval of the mean.

The listeners EEG responses were segmented in the −200 to 1000 ms interval surrounding the onset of the expected or unexpected word, generating an ERP. Figure 3a demonstrates the ERP to unexpected and expected words, and the distribution of individual subject amplitudes for the negative and positive peak. The mean negative peak amplitude was reduced 215.32% (from −0.22 to −0.69 microvolts) in the “unexpected” condition compared to the “expected” condition, and this difference was statistically significant (T = 3.59; p = 0.001; df = 17) (Fig. 3b). The mean positive amplitudes did not statistically differ between the two conditions (T = 0.89; p = 0.383; df = 17) (Fig. 3c). The average ERP to the expected and unexpected words are indicated in Fig. 3d for midline electrodes Cz, CPz, Pz, and P0z.

### Relationships between speaker spectra and listener ERP

**Figure 4:**
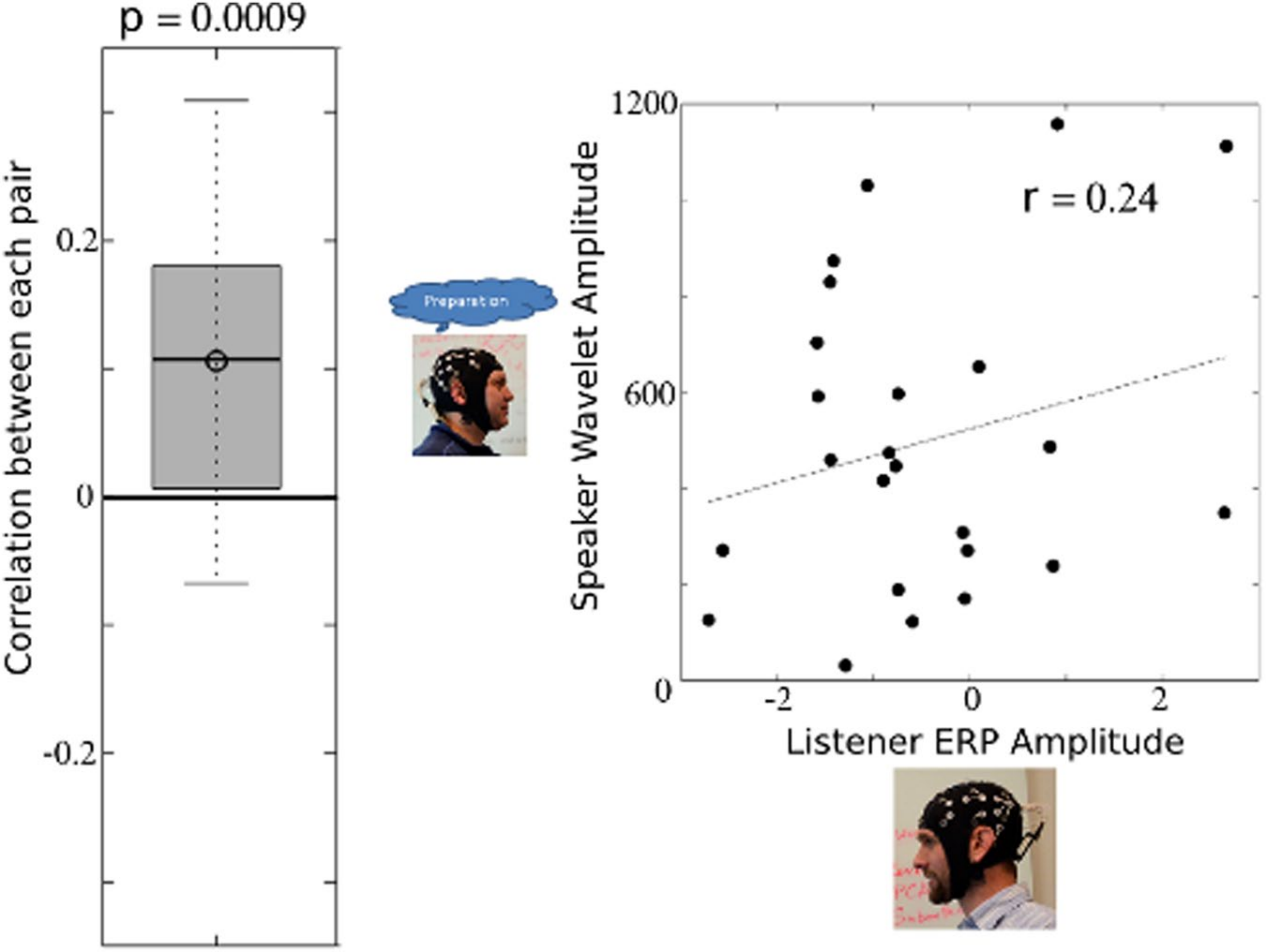
Relationships between speaker alpha and the listener ERP. The speaker’s preparatory alpha band (centered on 8 Hz) amplitudes were correlated with the listener’s minimum ERP amplitudes. The distribution of correlations across subject pairs is indicated in the boxplot in (**a**). The Fisher z-transformed Pearson correlation coefficients significantly differed from zero (p = 0.0009). A representative scatterplot of single-trial lower alpha band amplitudes within the speaker (y-axis) and single-trial listener ERP amplitudes (x-axis) is indicated in (**b**). Reduced wavelet amplitudes within the speaker during preparation appear to correspond to a greater N400 amplitude within the listener (r = 0.24). The 95% confidence interval includes r = −0.24 to r = 0.62).

For each subject pair, the speaker’s peak wavelet amplitudes (during preparation) were correlated with the listener’s positive and negative peak ERP amplitude to the unexpected word. Fisher z-transformed Pearson correlation coefficients between speaker amplitudes and listener single trial amplitudes were significantly greater than zero for the speaker’s lower alpha (8 Hz) amplitudes and the listener’s negative ERP peak to the unexpected word (T = 4.02; p = 0.0009; df = 17), with an average correlation of 0.1063 (std = 0.1123), and positive correlations present in 15 out of 18 subject pairs. The relationship between the speaker’s lower alpha (8 Hz) amplitudes and the listener’s negative ERP peak to the expected word did not reach statistical significance (T = 0.80; p = 0.4331; df = 17). Figure 4a demonstrates the distribution of subject pair correlation values, and Fig. 4b displays a scatterplot of single trial amplitudes between speaker (y-axis) and listener (x-axis) for an exemplar pair. Supplementary Fig. 2 indicates the 95% confidence intervals for each subject pair along with the number of trials used to compute correlations for each pair. The p-values of all 12 statistical tests (6 frequencies ×2 peaks) are indicated within Table 1.

**Table 1:** P-values of T-tests conducted to determine whether Fisher z-transformed Pearson correlation coefficients between speaker and listener pairs significantly differed from zero.

Speaker	Listener	
	Min. ERP peak	Max. ERP peak
2 Hz	0.5101	0.4285
6 Hz	0.4699	0.6787
8 Hz	0.0009*	0.0697
12 Hz	0.7405	0.3393
20 Hz	0.5509	0.2294
40 Hz	0.3634	0.6198

## Discussion

Within the present study, we demonstrate that decreases in the speaker’s lower alpha band responses during speech preparation are related to more negative listener N400 responses to the spoken word. These findings are specific to the lower alpha band frequency within the speaker, and the N400 response within the listener, since we were unable to demonstrate statistically significant relationships between the other frequencies or the positive ERP peak (i.e. the P600) which followed the N400. As far as we are aware, this is the first study to demonstrate an N400 response to unexpected words generated spontaneously by another individual, and this study demonstrates an approach to identify relationships among well-established EEG phenomenon between two individuals within a hyperscanning context.

The origins of the EEG alpha band are not entirely certain, but alpha activity is generally thought to emerge from visual areas when individuals are disengaged from visual tasks. For example, increases in alpha activity are observed when individuals close their eyes, and reductions in alpha activity are related to an increased ability to detect visual targets^18,23,32^. Consistent with the present study, previous studies have demonstrated alpha suppression during speech preparation directly^33^.

In the context of the present study, we hypothesize that reductions in alpha activity following the “expected” or “unexpected” cue correspond to more efficient processing of visual content that subsequently appeared (i.e. the sentence with an incomplete ending)^34^, resulting in an improved ability to come up with an unexpected sentence ending (i.e. a more negative N400 amplitude within the listener). This finding carries important implications for subsequent hyperscanning communication studies. For example, changes in alpha activity may serve as a useful measure of additional aspects of speech, such as the listener’s engagement^35^ or comprehension^36^ of the verbal content of communication. In addition, subsequent studies may determine whether reductions in alpha during speech preparation are related to additional EEG features that track the listened speech, such as the degree in which the listeners EEG signal tracks the speech envelope^37^.

There are a few features of the N400 response within the present study which are important to note. First, the latency of the negative peak appears earlier than 400 ms, consistent with previous studies using continuous speech stimuli^7,15^. However, the onset timing of the N400 within the present study (~200 ms) and the slightly right lateralized central-parietal topography (Fig. 3a) is consistent with the classic N400 effect observed for written and spoken stimuli^1,8^. Thus, the timing and topography of the N400 to spontaneous unexpected spoken words appears qualitatively similar to the N400 effect in traditional experimental settings.

It is interesting to note the presence of a P600 response in the present study, despite the greater sensitivity of the P600 to syntactic vs. semantic violations. This finding emphasizes that the P600 may be modulated by additional factors than syntax alone^38^, and raises the possibility that the P600 (and the earlier N400 peak onset) may have appeared due to the greater biological relevance of listening to a speaker spontaneously generating words in real time.

The lack of a statistical relationship between listener P600 amplitudes and the speakers preparatory alpha activity suggests that error repair processes^13,14^ may be unrelated to the processes that contribute to speech preparation (i.e. effort) within the speaker. Alternatively, the relationship between listener N400 amplitudes and the speaker’s preparatory alpha activity may appear since each process overlaps in their sensitivity to individual effort and cortical processing. For example, according to predictive coding theories, larger negative N400 amplitudes reflect the passing of unexpected information through the cortical hierarchy^11^. Thus, more negative N400 responses reflect the greater amount of processing resources that are required when speech violates predictions. Thus, increases in effort or cortical processing within the speaker (corresponding to reductions in alpha activity) are related to increased effort and processing within the listener in understanding the produced content.

There are a few limitations to the present study which should be considered. Due to the spontaneous nature of the experiment, it is difficult to control the different categories of unexpected sentence endings that are produced. For example, the speaker could potentially produce an unexpected sentence ending that was within the same category as the expected word (e.g. I took my dog for a stroll), or an unexpected sentence ending that was within a difference category (e.g. I took my dog for a drink). N400 amplitudes increase when the unexpected word is both unexpected and within a different category from the expected word^39,40^, so subsequent studies with a greater number of trials could potentially distinguish between responses to these different sentence categories.

In addition, N400 amplitudes could potentially differ within the “unexpected” condition in the present study, since the N400 is reduced when the preceding words show greater semantic association with the final words (Kutas & Van Petten, 1988)^41^. Differences in the prosody of the final word could modulate N400/P600 amplitudes^42^, and the repetition of sentences within the present experiment (i.e. each sentence being present within the “expected” and “unexpected” conditions for each block) could also account for some of the variance in the N400/P600 amplitudes that were measured.

Since there is considerable noise in computing correlations from single-trial amplitudes, increasing the number of participants and trials could additionally improve the ability to identify statistically significant effects, including improving the ability to distinguish whether the absence of a relationship between lower alpha band responses and the P600 is due to a lack of statistical power, or differences between the degree in which the N400 and P600 responses are modulated by the experimental stimuli. For example, the N400 appears more directly modulated by semantic incongruities, while the P600 response generally appears to reflect the reprocessing of syntactic anomalies^12,43,44^. In addition, the differences in amplitudes observed in the present study (derived by identifying the peak time point of the overall average) may appear either due to a decreased amplitude of the N400 component, a change in latency of the N400, a change in its shape, or a combination of these factors^45^.

The hyperscanning context introduces important additional considerations which can influence the results. While the speakers within the present study were instructed to minimize facial expressions (to reduce motion artifacts), it is possible that subtle visual or auditory cues appeared following the task instruction or following the appearance of the sentence, which may have prepared the listener for an expected or unexpected sentence ending. In this circumstance, the listener could potentially anticipate an unexpected sentence ending, which would result in a reduced N400 effect.

The present study may motivate further research aimed at understanding ERP responses that appear within complex social environments. For example, the P300 ERP response is robustly present following rare tones that appear within a series of frequent tones^46,47^. The response is thought to represent a reorganization of brain networks involved in representing and responding appropriately to the unexpected event^48^. However, it is unclear whether this canonical P300 response is observed within complex social settings (e.g. when the phone rings or when someone calls your name), or whether a fundamentally different response appears within such settings. The same question is important to ask of the N400 response. However, the spatial topography and time course of the N400 in the present study appears qualitatively similar to the canonical N400, suggesting that similar processes may emerge within traditional experimental contexts and our extemporaneous social setting.

The N400 effect is generally observed following semantic incongruity, with the semantic incongruity often presented visually as a series of words on a computer screen, or aurally, as a series of recorded words through speakers^1^. However, the N400 response has also been observed to more complicated incongruities, such as viewing incorrect mathematical equations (e.g. 8 × 5 = 35)^49^, or viewing unexpected actions^50^. In addition, the N400 effect has been observed using pictures^51^, American Sign Language, pseudowords, and lists of items^41,52^. As far as we are aware, the present study is the first to demonstrate the presence of an N400 response to unexpected sentence endings that were generated extemporaneously and spoken aloud in real-time to the listener. The presence of an N400 response in this context is encouraging with respect to our abilities to leverage this ERP component, as well as other well-defined ERP components such as the P300 and P600, to understand brain processes in social contexts.

## Electronic supplementary material

Supplementary Information

## Data Availability

The de-identified data will be publicly available within COINS open source software (http://coins.mrn.org/dx).
