# Supplementary material for: Relationships between alpha oscillations during speech preparation and the listener N400 ERP to the produced speech
Source: Sci Rep. 2018 Aug 27;8:12838. doi: 10.1038/s41598-018-31038-9 (PMC6110750; doi:10.1038/s41598-018-31038-9)
Supplement: Supplementary file 1 — Supplementary Information [file 41598_2018_31038_MOESM1_ESM.pdf]

Supplementary Material: Relationships between alpha oscillations during speech preparation and the listener N400 ERP to the produced speech

David A. Bridwell, Sarah Henderson, Marieke Sorge, Sergey Plis, Vince D. Calhoun

Supplementary Table 1: Sentence Stimuli and Cloze Probability of the Frequent Word.

Sentence	Frequent Word (expected)	Cloze Probability (expected)	Cloze Probability (unexpected)
For Thanksgiving dinner, we cooked a _____.	Turkey	1.000	0.063
The dog heard a noise and began to _____.	Bark	1.000	0.067
The chicken laid an _____.	Egg	1.000	0.071
At the pumpkin patch we picked out a _____.	Pumpkin	1.000	0.118
Curiosity killed the _____.	Cat	1.000	0.118
The best part of waking up, is Folgers in your _____.	Cup	1.000	0.133
Spiders have eight _____.	Legs	1.000	0.154
The grass is _____.	Green	1.000	0.267
The cow said _____.	Moo	1.000	0.313
You can't judge a book by it's _____.	Cover	0.941	0.118
For his magic trick, he pulled a rabbit out of a _____.	Hat	0.938	0.071
In the Fall, the leaves fall off of the _____.	Trees	0.938	0.125
I was thirsty, so I took a drink of _____.	Water	0.938	0.286
I took my dog for a _____.	Walk	0.933	0.059
He couldn't afford a new car, he needed more _____.	Money	0.929	0.077
The sky is _____.	Blue	0.929	0.143
At Christmas, Santa came down the _____.	Chimney	0.929	0.182
I ate the soup with a _____.	Spoon	0.917	0.125
To celebrate my birthday we ate _____.	Cake	0.882	0.077
There was a test, so he spent the weekend _____.	Studying	0.875	0.188
When you are sick, it helps to visit the _____.	Doctor	0.867	0.125
In the Spring, the flowers begin to _____.	Bloom	0.857	0.133
She hated swimming, she was afraid of the _____.	Water	0.824	0.125
When he was five, I taught him to tie his _____.	Shoes	0.786	0.200
They used treats to train their new _____.	Dog	0.769	0.143
I pulled the mail out of the _____.	Mailbox	0.750	0.167
To listen to music, I turned on the car's _____.	Radio	0.750	0.333
I ran five miles a day to train for the _____.	Marathon	0.733	0.083
At work I sit at a _____.	Desk	0.733	0.143
She was really tired, so she went to _____.	Bed	0.714	0.133
I started the lawn mower and mowed the _____.	Lawn	0.714	0.167
To greet his owner, the dog began to _____.	Bark	0.692	0.143
The Television show was interrupted by a _____.	Commercial	0.688	0.188
The sun was in her eyes so she put on a pair of _____.	Sunglasses	0.667	0.308
He sat down on the _____.	Chair	0.636	0.143
Instead of driving to Oregon, they decided to _____.	Fly	0.615	0.235
It was cold outside so I wore a _____.	Jacket	0.533	0.118

At checkout, she reached into her purse for a _____.	Credit card	0.438	0.118
We visited the zoo to see the _____.	Lion	0.438	0.214
There were crumbs on the floor, so he got a _____.	Broom	0.400	0.182

Supplementary Methods- Instructions. At the onset of the experiment, the speaker was instructed as follows: “During the experiment, you will see a prompt for “humorous” or “serious”, followed by a sentence with the final word missing. During the “humorous” prompt you should fill in the missing word with something that is “humorous” and during the “serious” prompt you should fill in the missing word with something that is “serious”. For example, if the sentence is “I took my dog for a blank”, during the “humorous” condition you might say “I took my dog for a unicorn” and during the “serious” condition you might say “I took my dog for a walk. In most cases, the “humorous” ending won’t actually be humorous, but we want to encourage you to come up with something that is unexpected or creative.”

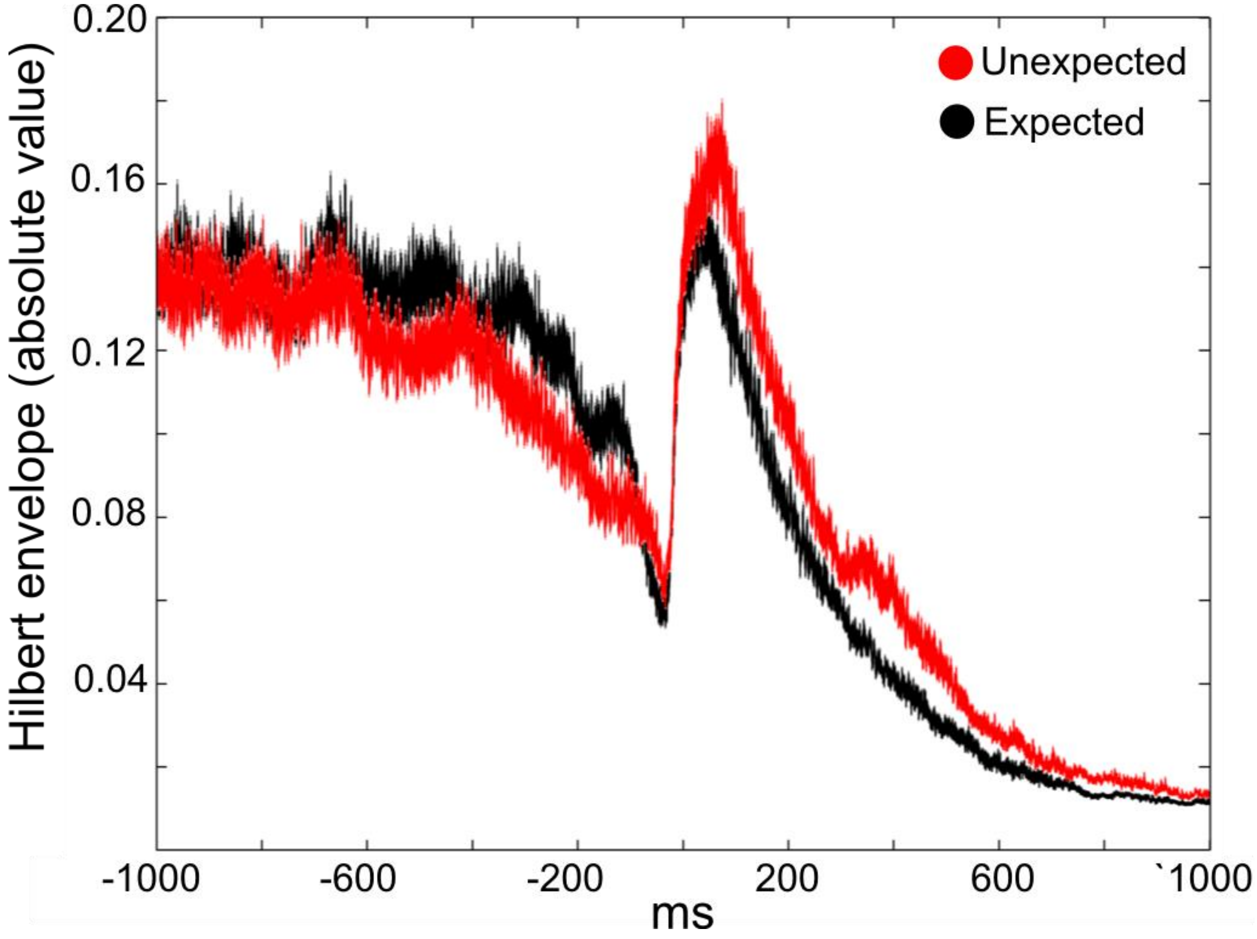

Supplementary Figure 1. Average speech envelope following the expected or unexpected sentence ending. The absolute value of the Hilbert envelope was calculated and averaged across subjects for the “expected” (black) and “unexpected” (red) conditions. There is a clear drop in spectral energy around 0 ms which corresponds to the onset of the final word. The timing of the peak decrease in spectral energy around 0 ms and the peak increase that follows appear similar. Since the sentence was spoken continuously, the final word immediately followed the prior words in the sentence. As a result, the speech envelops is elevated in the interval preceding the onset of the final word.

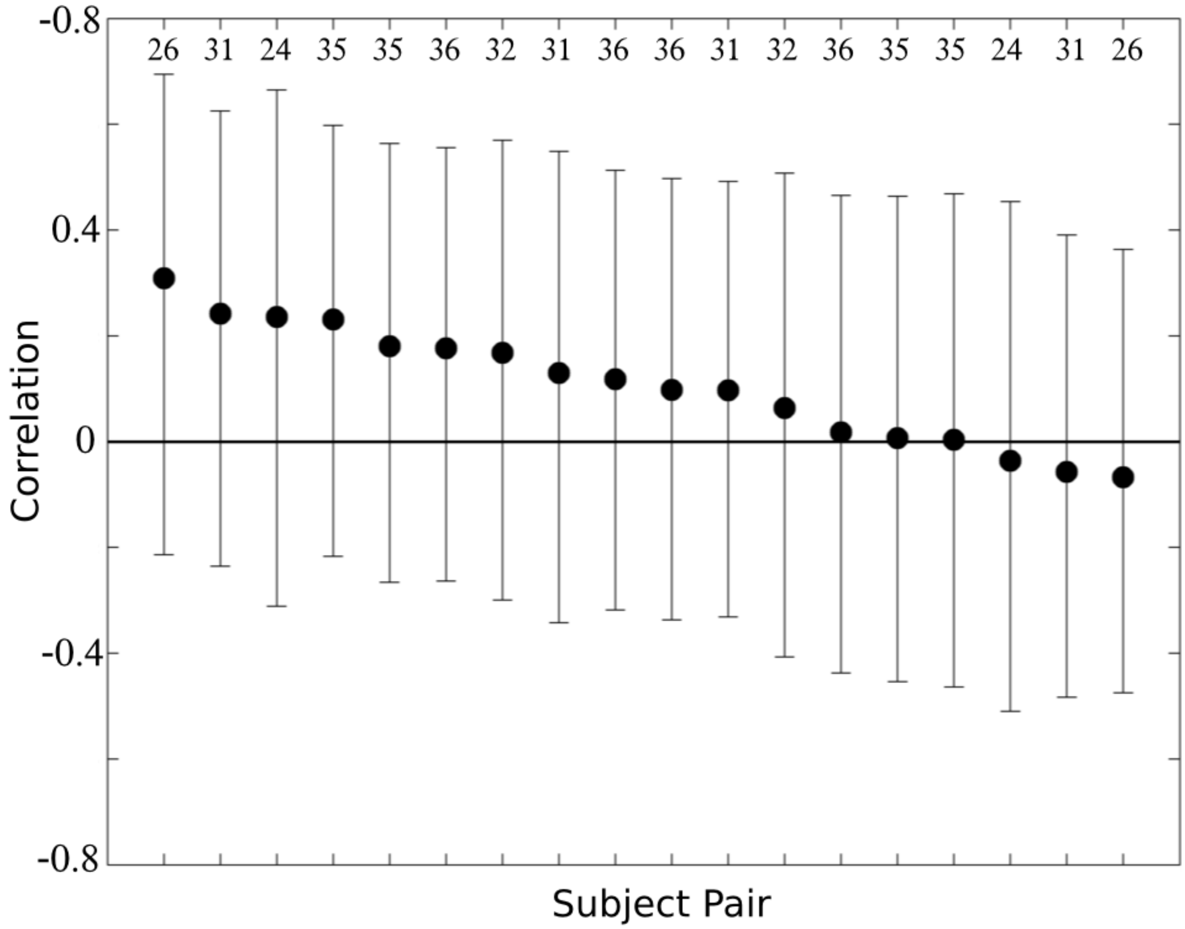

Supplementary Figure 2. The Pearson correlation coefficient (y-axis) between the speakers lower alpha band power and listeners N400 amplitude are indicated for each subject pair (x-axis). Errorbars indicate the 95% confidence interval and the number of trials used within each calculation are indicated at the top of the plot.
